# Physical Comorbidity and Health Literacy Mediate the Relationship Between Social Support and Depression Among Patients With Hypertension

**DOI:** 10.3389/fpubh.2020.00304

**Published:** 2020-08-05

**Authors:** Baiyang Zhang, Wenjie Zhang, Xiaxia Sun, Jingjing Ge, Danping Liu

**Affiliations:** ^1^West China School of Public Health and West China Fourth Hospital, Sichuan University, Chengdu, China; ^2^The Department of Academic Affairs, West China School of Medicine/ West China Hospital, Sichuan University, Chengdu, China

**Keywords:** social support, physical comorbidity, health literacy, depression, hypertension

## Abstract

Depression is a common comorbidity among patients with hypertension. Patients with hypertension and depression have worse health outcomes compared to those without depression. The combined effects of social support, physical comorbidity, and health literacy on depression among individuals with hypertension remain unclear. A survey was conducted between December 2017 and May 2018 to investigate the relationships among social support, physical comorbidity, health literacy, and depression in a population of patients with hypertension in rural areas of Sichuan province, China. Multiple linear regression was used to examine factors that influenced depression, and structural equation modeling (SEM) was used to examine the relationships among the four study variables. The mean scores of 549 patients with hypertension were 37.17 ± 6.84 for social support, 14.62 ± 6.26 for health literacy, and 3.56 ± 3.05 for depression; furthermore, 34.2% of participants had physical comorbidity. Gender and per capita annual family income were significantly associated with depression. Physical comorbidity was directly positively related to depression while health literacy was directly negatively related to depression. Social support had an indirect negative association with depression by the mediating effects of health literacy and physical comorbidity. Adequate social support and health literacy, and less physical comorbidity could potentially contribute to reducing depression. The study highlights the importance of social support in maintaining mental health among patients with hypertension. Strategies that target the enhancement of social support and health literacy should be prioritized to relieve depression among patients with hypertension. More attention should be paid to women, low-income individuals, and patients with physical comorbidities.

## Introduction

Hypertension is one of the most severe chronic diseases and a leading cause of mortality and disability, causing almost 10 million deaths worldwide in 2013 and accounting for 7% of global Disability Adjusted Life Years lost (1). By 2025, ~29% of the world's population is expected to have this disease. In China, successive population surveys have reported an increasing prevalence of hypertension, with the disease affecting 18.8% of the population in 2002 (2) and 27.8% in 2014 (3). Given the rapidly increasing prevalence of hypertension and the burden of disease, its timely and effective control and management has become a basic public health service priority in China.

Depression is recognized as the fourth leading contributor to the global burden of disease (4). By 2020, the burden of depression is projected to account for 5.7% of the total disease burden (1). Clinical depression or depressive disorder is a common comorbidity among individuals with hypertension and epidemiological studies have demonstrated an increased co-occurrence of depression with hypertension (5). Depressive mood is a risk factor for the development of high blood pressure and is known to increase the occurrence of uncontrolled hypertension (6, 7). Patients with hypertension and depression have worse health status, poorer quality of life, impaired well-being, higher health care expenditure, and increased mortality compared to those without depression (7–10). In addition, depression can mask or mimic the symptoms of chronic medical illnesses and anti-depression medications may interact pharmacologically with anti-hypertensive medications, complicating treatment and resulting in poor prognosis (11). These findings emphasize the importance of addressing depression in patients with hypertension.

Social support refers to the “social resources that persons perceive to be available or that are provided to them.” Prior studies have demonstrated the association between inadequate social support and depression among patients with hypertension (12–14). Poor social support has also been linked to poor adherence to anti-hypertension treatment and poor blood pressure control (15–17) and may thus cause poor prognosis and eventually affect the mental state of patients with hypertension. In a study of Korean elderly patients with hypertension, both social support and depression were influencing factors of self-care behavior (18).

Physical comorbidity refers to a person suffering from two or more physical diseases at the same time. Physical comorbidity appears to have an impact on depression. In the general population, somatic comorbidity is associated with depression (19). The presence of comorbid chronic diseases is associated with depressive symptoms in older patients with hypertension (12). In addition, persistent depression is significantly more likely to occur in veterans with hypertension and multi-morbidity than in those with only hypertension (20). Health literacy is defined as the “degree to which individuals have the capacity to obtain, process, and understand basic health information and services needed to make appropriate health decisions” (21). Previous studies have demonstrated that poor literacy is associated with higher levels of depressive symptoms in populations of US smokers with low socioeconomic status, US adults with addictions, and Korean adults (22–24). Additionally, a study of patients with diabetes indicated that depression may reduce the positive effect of health literacy on self-management (25). Among patients with hypertension, associations between health literacy and medication adherence, hypertension management and control, clinical outcomes (e.g., systolic and diastolic blood pressure), and health-related quality of life have been noted (26–30). However, the relationship between health literacy and depression among patients with hypertension remains unclear.

The prevalence of hypertension in China is increasing more rapidly in rural areas than in urban areas (31). This tendency is related to the adoption of urban lifestyles due to economic development, along with improved diagnostics (32, 33). Due to a lack of contact opportunity, as well as their lower financial and education level, rural individuals with hypertension have poor social support and health literacy (34, 35). Further, hypertension has been effectively controlled in a low percentage of these patients (31), thereby increasing their difficulty in coping with depressive symptoms and the possibility of developing other physical comorbidities. This study focuses on patients with hypertension in rural areas.

Most previous studies examined the associations between social support, physical comorbidity, health literacy, and depression; however, the combined effects of these factors on depression and the underlying mechanisms of those relationships remain unclear. Social support has been positively associated with health literacy among patients with chronic kidney disease and patients with coronary heart disease (36, 37). The mediating role of health literacy between subjective social status and depressive symptoms has also been noted (38). Therefore, we speculated that health literacy may be a potential mediator between social support and depression among hypertensive patients. In addition, prior research has demonstrated the relationship between lower social support and increased comorbidity, as well as the interactions of social support and multiple physical chronic conditions in explaining depression among the elderly (39, 40). Thus, we hypothesized the mediation effect of physical comorbidity on the association between social support and depression among hypertensive patients. Moreover, health literacy has been shown to be independently related to disease knowledge (41). Suffering from physical comorbidity may increase hypertension patients' knowledge of multiple diseases, thus potentially improving their health literacy.

**Figure 1:**
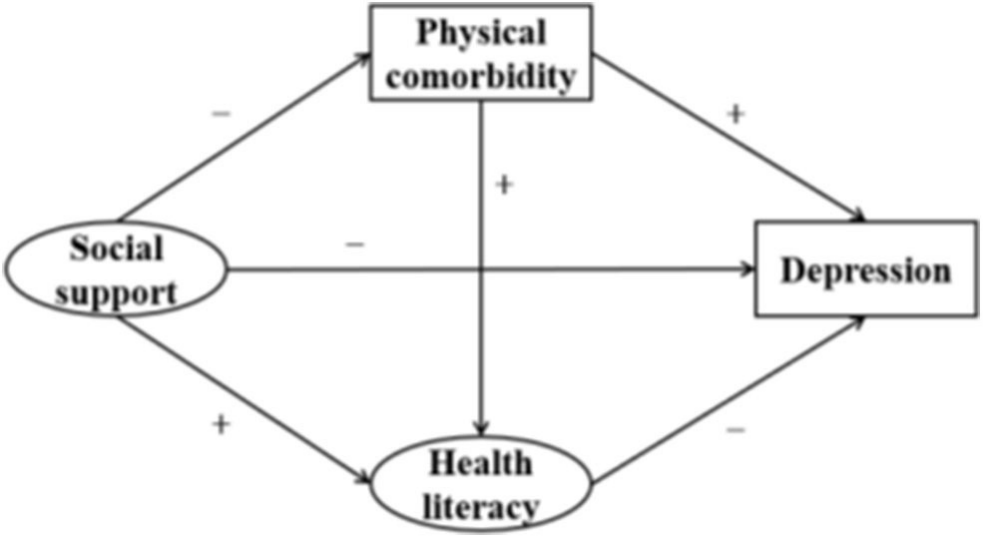
Theoretical model and hypotheses.

Structural equation modeling (SEM) is an ideal data analytic technique to study the interrelationships between latent variables, which cannot be measured directly. These include social support, health literacy, and depression in the current research. Additionally, this model can be used to test the mediating effect by path analysis (42). We constructed a structural equation model to explore the association between social support and depression, as well as to examine whether this relationship could be explained by the mediation effects of physical comorbidity and health literacy in patients with hypertension. We hope to eventually provide reference for interventions targeting the improvement of the mental health of patients with hypertension. According to the theoretical framework mentioned above, we developed the hypotheses shown in Table 1, which correspond to the structural equation model shown in Figure 1.

**Table 1:** The theoretical hypotheses.

1. Social support has a direct negative relationship with depression.
2. Physical comorbidity has a direct positive relationship with depression.
3. Health literacy has a direct negative relationship with depression.
4. Social support has a direct negative relationship with physical comorbidity.
5. Social support has a direct positive relationship with health literacy.
6. Physical comorbidity has a direct positive relationship with health literacy.
7. The relationship between social support and depression is mediated by physical comorbidity.
8. The relationship between social support and depression is mediated by health literacy.
9. The relationship between social support and health literacy is mediated by physical comorbidity.
10. The relationship between physical comorbidity and depression is mediated by health literacy.

## Materials and Methods

### Sample

We conducted a cross-sectional study in Sichuan Province, China, between December 2017 and May 2018. The sample size was calculated using the formula: $\text{n}={\left(\frac{\text{Z}\alpha \text{/2}}{\delta }\right)}^{2}\times \pi \times \left(1-\pi \right)$; π = 5.7%, the depression rate of patients with hypertension in a community-based study conducted in 2014 (43), δ = 2%, α = 0.05, and *Zα*/2 = 1.96. According to the formula, the sample size was calculated as 516. Considering possible dropout, we increased the sample size by 20% to 600. We randomly selected a city in Sichuan Province and 10 townships in rural areas of the city were randomly selected as survey areas. We used systematic sampling to obtain 60 subjects from a database of patients with hypertension established by each township hospital. Therefore, 600 patients with hypertension were interviewed face to face by professionally trained investigators. To ensure the quality of the investigation, we set the following exclusion criteria: (1) patients who could not properly answer questions due to physical disability or cognitive impairment, and (2) those unable to cooperate for personal reasons (they had the freedom to withdraw without consequence). According to these standards, 549 (91.5%) valid responses were ultimately analyzed. All of the participants signed to convey their informed consent before the investigation and were voluntary in their participation. The ethical approval of the data collection was given by the ethics committee of Sichuan University.

### Instruments

The questionnaire included five domains: socio-demographics, social support, physical comorbidity, health literacy, and depression.

#### Socio-Demographics

Socio-demographics consisted of gender, age, education, marital status, per capita annual household income, and living arrangements. Age was categorized as <60, 60–69, 70–79, and ≥80 years. Educational level was divided into “no formal education,” “primary school,” and “middle school and above.” Marital status was defined as a binary variable: “married with spouse” and “divorced, widowed, or unmarried.” Per capita annual household income was classified into three categories: <$750, $750–1499, and ≥$1500. Living arrangements were defined as “living with family members” or “living alone.”

#### Social Support

Social support was assessed through The Social Support Rating Scale (44). This scale was specifically designed for use in a Chinese context. It contains three subscales: subjective support (the level of perceived support), objective support (the level of actual or visible support), and support utilization (the degree to which available support was used). Subjective support is assessed via four items, with a possible score range of 8–32; objective support is assessed via three items, with a possible score range of 1–22; and support utilization is assessed via three items, with a possible score range of 3–12. The total score of the scale ranges from 12 to 66, whereby higher scores reflect better social support. It is generally considered that a score from 12 to 22 indicates a low level of social support, 23–44 indicates moderate support, and 45–66 indicates high support (45). This scale had a reported Cronbach's α coefficient of 0.89 and a test–retest reliability of 0.92 in prior research (44).

#### Health Literacy

A modified version of The Chinese Citizen Health Literacy Questionnaire, developed by the National Health Commission of the People's Republic of China, was used to assess health literacy. Representative questions related to health literacy among patients with hypertension were selected by experts. To improve the study questionnaire, we added questions addressing individuals' knowledge of the prevention and control of common chronic disease. The questionnaire contained three dimensions: knowledge and belief literacy, behavior literacy, and skill literacy, with 33 items and a total possible score of 33. Respondents who correctly answered 80% or more of the questions were regarded as having good health literacy (46). In the current study, this scale has a Cronbach's α coefficient of 0.864.

#### Physical Comorbidity

The physical comorbidity data concerned physical conditions that had been diagnosed by a health professional and that were expected to persist or had already persisted for 6 months or more. The number of physical comorbidities other than hypertension was calculated and categorized as 0, 1, or 2+.

#### Depression

Depression was measured using the Centre for Epidemiologic Studies Depression Scale 10-item version (CESD-10), which has been demonstrated to appropriately reflect depressive symptoms experienced in the previous week (47). The CESD-10 includes three items addressing depressed affect, five items addressing somatic symptoms, and two items addressing positive affect. Options for each item range from “rarely or none of the time” (score of 0) to “all of the time” (score of 3). Scoring is reversed for items 5 and 8, which are positive affect statements. Total scores can range from 0 to 30. Scores of 10 or over indicate clinically relevant depression (47). The scale has excellent internal reliability (Cronbach's α = 0.80) and good validity (48).

### Data Management and Analysis

A database was established using EpiData Version 3.1, and statistical analyses were conducted using SPSS 21.0 and AMOS 20.0. First, descriptive statistics (frequencies, percentages, means, and standard deviations) were calculated to describe the sample. Next, we obtained Pearson correlations to explore the relationships among social support, physical comorbidity, health literacy, and depression. Multiple linear regression was used to estimate associations between the independent variables and depression. Finally, SEM was used to test the hypotheses. We used the subscale scores of social support and health literacy as measurement variables and the total scores of these measures as latent variables. Physical comorbidity and depression were included as measurement variables. Statistical significance was set at *P* < 0.05.

## Results

### Descriptive Statistics

Descriptive statistics of the sample are displayed in Table 2. Our sample contained 343 women (62.5%) and 206 men (37.5%). The highest proportion was 60–69 years old (41.2%), with a primary school education (52.1%). Most were married (78.0%) and lived with family members (90.0%). Most participants had a per capita annual household income in the $750–1499 range (47.0%).

**Table 2:** Descriptive results of the sample.

**Variable**	***N* (%), Mean ± SD**
Gender	
Men	206 (37.5)
Women	343 (62.5)
Age (years)	
<60	87 (15.8)
60–69	226 (41.2)
70–79	188 (34.3)
≥80	48 (8.7)
Education	
No formal education	123 (22.4)
Primary school	286 (52.1)
Middle school and above	140 (25.5)
Marital status	
Married have spouses	428 (78.0)
Divorced, widowed, or unmarried	121 (22.0)
Per capita annual household income, $	
<750	124 (22.6)
750–1499	258 (47.0)
≥1500	167 (30.4)
Living arrangements	
Living with family members	494 (90.0)
Living alone	55 (10.0)
Physical comorbidities	
0	361 (65.8)
1	138 (25.1)
2~	50 (9.1)
Type 2 diabetes mellitus	93 (34.7)
Chronic tracheitis/bronchitis	35 (13.1)
Hyperlipemia	22 (8.2)
Chronic gastrointestinal diseases	21 (7.8)
Hyperosteogeny	20 (7.5)
Coronary disease	19 (7.1)
Lumbar disc protrusion	19 (7.1)
Others	39 (14.5)
Social support	37.17 ± 6.84
Subjective support	22.79 ± 2.42
Objective support	7.91 ± 2.42
Support utilization	6.47 ± 2.08
Health literacy	14.62 ± 6.26
Knowledge and belief literacy	8.60 ± 4.83
Behavior literacy	4.79 ± 1.71
Skill literacy	1.23 ± 0.53
Depression	3.56 ± 3.05

The mean scores for social support, health literacy, and depression were 37.17 ± 6.84, 14.62 ± 6.26, and 3.56 ± 3.05, respectively. The proportion of individuals with a high level of social support was 16.9%, and only 5.6% of the subjects had adequate health literacy. In addition, 6.0% of the patients with hypertension had depressive symptoms. The proportion of participants with physical comorbidity was 34.2%. Type 2 diabetes mellitus ranked first in the number of physical comorbidities. The percentage of people with various physical comorbidities is also displayed in the table.

### Correlations Between Study Variables

Correlations between key variables are presented in Table 3. Social support was positively correlated with health literacy but negatively correlated with physical comorbidity and depression. There was a significant positive correlation between physical comorbidity and health literacy and depression. Health literacy was significantly negatively correlated with depression.

**Table 3:** The correlation among key variables.

	**Social support**	**Physical comorbidity**	**Health literacy**	**Depression**
Social support				
Physical comorbidity	−0.207^**^			
Health literacy	0.135^*^	0.127^*^		
Depression	−0.147^*^	0.256^**^	−0.225^**^	

* P < 0.01,

** *P < 0.001*.

### Linear Regression Analysis of Study Variables

Table 4 reveals that depression among patients with hypertension was associated with the two socio-demographic factors of gender and per capita annual household income, in addition to physical comorbidity and health literacy. The depressive symptoms of women with hypertension were more serious than those of men (β = 0.725, *P* = 0.005). Compared with the <$750 group, subjects with hypertension with a per capita annual household income of ≥$1500 had fewer depressive symptoms (β = −0.723, *P* = 0.037).

**Table 4:** Multiple linear regression analysis of factors associated with depression.

	**β**	***t***	***P***	**95% CI**
Constant	4.295	3.462	0.001	1.858, 6.732
Gender				
Men (ref)				
Women	0.725	2.811	0.005	0.219, 1.232
Age				
<60 (ref)				
60–69	−0.104	−0.283	0.777	−0.827, 0.619
70–79	0.593	1.574	0.116	−0.147, 1.334
≥80	0.147	0.272	0.785	−0.911, 1.205
Education				
No formal education (ref)				
Primary school	0.533	1.614	0.107	−0.116, 1.181
Middle school and above	0.554	1.423	0.155	−0.211, 1.319
Marital status				
Married have spouses (ref)				
Divorced, widowed, or unmarried	0.509	1.497	0.135	−0.159, 1.176
Per capita annual household income, $				
<750(ref)				
750–1,499	−0.460	−1.445	0.149	−1.086, 0.165
≥1,500	−0.723	−2.088	0.037	−1.404, −0.043
Living arrangements				
Living with family members (ref)				
Living alone	−0.553	−1.176	0.240	−1.477, 0.371
Physical comorbidity				
0 (ref)				
1	1.316	4.500	0.000	0.741, 1.890
≥2	2.528	5.756	0.000	1.665, 3.390
Social support	−0.025	−1.215	0.225	−0.066, 0.016
Health literacy	−0.116	−5.479	0.000	−0.158, −0.075

### Test of Study Model

**Figure 2:**
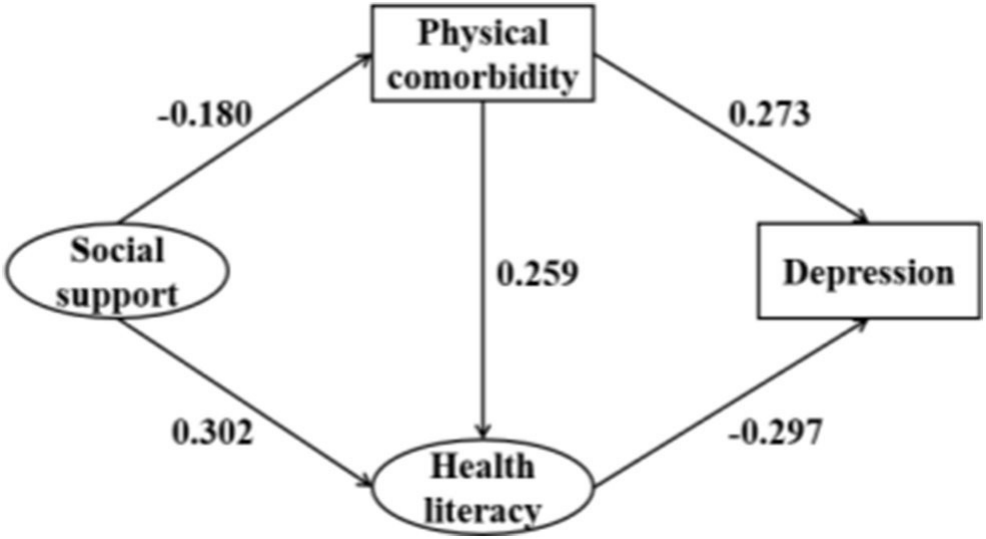
Structural analysis of social support, physical comorbidity, health, literacy, and depression. All coefficients are significant (*P* < 0.05).

We used SEM to test the model shown in Figure 1. The path coefficient of the link between social support and depression was not statistically significant. Thus, we revised the model by removing this path. After setting socio-demographic characteristics as covariates, the direction of influence among the key variables remained unchanged and the corresponding coefficients did not change significantly. Thus, the socio-demographic characteristics were not confounding factors and were not considered in the final model. To improve the model fitness, the covariance between measurement errors was set based on the modification indices. Figure 2 shows the final modified model that tested the associations of social support, physical comorbidity, and health literacy with depression. Standardized coefficients representing the direct associations between variables are displayed over the arrows. The model demonstrated good fit: RMSEA = 0.062, TLI = 0.931, CFI = 0.958, χ^2^/df = 3.117.

The direct, indirect, and total effects of key study variables are displayed in Table 5. Physical comorbidity had a direct effect (β = 0.273, 95% CI: 0.219–0.341) on the depression of patients with hypertension, thus supporting Hypothesis 2. Health literacy was directly associated with depression (β = −0.297, 95% CI: −0.381 to −0.197), thus supporting Hypothesis 3. However, social support was only indirectly associated with depression (β = −0.125, 95% CI: −0.162 to −0.077), rather than directly associated, leading us to reject Hypothesis 1. Greater social support was associated with reduced likelihood of having physical comorbidity (β = −0.180, 95% CI: −0.271 to −0.074) and greater health literacy (β = 0.302, 95% CI: 0.188–0.402), thus supporting Hypotheses 4 and 5. Physical comorbidity had a direct association with the health literacy of patients with hypertension (β = 0.259, 95% CI: 0.208–0.319), thus supporting Hypothesis 6.

**Table 5:** Direct, indirect, and total effects of key study variables.

**Model pathways**	**Standardized coefficient**	**95% CI**
**Total effects**		
Social support → Physical comorbidity	−0.180	−0.271 to −0.074
Social support → Health literacy	0.255	0.146 to 0.366
Social support → Depression	−0.125	−0.162 to −0.077
Physical comorbidity → Health literacy	0.259	0.208 to 0.319
Physical comorbidity → Depression	0.196	0.136 to 0.262
Health literacy → Depression	−0.297	−0.381 to −0.197
**Direct effects**		
Social support → Physical comorbidity	−0.180	−0.271 to −0.074
Social support → Health literacy	0.302	0.188 to 0.402
Physical comorbidity → Health literacy	0.259	0.208 to 0.319
Physical comorbidity → Depression	0.273	0.219 to 0.341
Health literacy → Depression	−0.297	−0.381 to −0.197
**Indirect effects**		
Social support → Health literacy	−0.047	−0.074 to −0.016
Social support → Depression	−0.125	−0.162 to −0.077
Physical comorbidity → Depression	−0.077	−0.112 to −0.048

The results of significance testing of the mediating pathways are displayed in Table 6. A mediating effect was considered statistically significant if the 95% confidence interval did not include zero. The results illustrated that the relationship between social support and depression was mediated by physical comorbidity and health literacy (95% CI: −0.160 to −0.045 and −0.282 to −0.097, respectively), thus supporting Hypotheses 7 and 8. In addition, physical comorbidity mediated the relationship of social support with health literacy (95% CI: −0.198 to −0.065), thus supporting Hypothesis 9. Health literacy mediated the relationship between physical comorbidity and depression (95% CI: −0.473 to −0.240), thus supporting Hypothesis 10.

**Table 6:** Significance tests of mediating pathways.

**Model pathways**	**95% CI**
Social support → Physical comorbidity → Depression	−0.160 to −0.045
Social support → Health literacy → Depression	−0.282 to −0.097
Social support → Physical comorbidity → Health literacy	−0.198 to −0.065
Physical comorbidity → Health literacy → Depression	−0.473 to −0.240

## Discussion

To the best of our knowledge, this study is the first to explore the relationships among social support, physical comorbidity, health literacy, and depression in patients with hypertension in China. Hypertension is typically controlled less well in rural residents than in urban residents because of differences in educational level, economic level, and other factors. Furthermore, rural patients with chronic disease tend to have a higher prevalence of depression (49). Depressive symptoms of patients with hypertension may aggravate their health status by lowering therapeutic compliance, limiting health care access, reducing social support, and increasing the incidence of uncontrolled hypertension and comorbidities (7). Thus, it is important to study the factors that affect depression among rural patients with hypertension. In this study, the mean depression score of rural patients with hypertension was 3.56, and 6.0% of patients with hypertension had symptoms of depression.

Physical comorbidities among individuals with hypertension are more common than among those with normal blood pressure (50). A continuous survey of Korean citizens demonstrated that hypertension has a positive association with the risk of comorbidities and patients with hypertension are more than twice as likely to have comorbidities as non-hypertensive adults (51). In the current study, 34.2% of the subjects had physical comorbidity. Previous studies have suggested that the risk of depression increases in the presence of physical illnesses such as myocardial infarct, cerebrovascular disease, and diabetes mellitus (52–54). In the current study, more physical comorbidities were associated with an increased risk of depression among patients with hypertension, which may have resulted from persistent limitations in daily functioning associated with the coexisting diseases. The same conclusion was drawn in another study, which showed an increased severity of depression in patients with hypertension and type 2 diabetes mellitus (55). In addition, comorbidity is not conducive to the control and prognosis of hypertension. A study showed that 60.1% of patients with hypertension and without diabetes achieved their blood pressure control target, compared with just 24.3% of patients with hypertension and diabetes (56).

In this study, the mean health literacy score of patients with hypertension was 14.62 and only 5.6% of the subjects had adequate health literacy, indicating that the health literacy of the sample was rather poor. This is unsurprising given our focus on a rural population, as prior work has revealed poor health literacy in rural patients with hypertension is relatively common (31, 57). A survey conducted in Heilongjiang Province also showed that health literacy, especially regarding hypertension knowledge, was extremely low in rural areas of China (58). In addition, older age and lower education levels have been shown to be associated with poor health literacy in patients with hypertension (59). In this study, the average age of the respondents was relatively old (67.7 ± 8.6 years), and their educational level was generally low (74.5% had an educational level of primary school and below), which may explain the poor health literacy. A lack of health literacy is known to cause poor adherence to medication regimens and, consequently, poor management of hypertension and poor blood pressure control (60); thus, it is essential to promote health literacy among rural patients with hypertension.

The present study represents the first exploration of the association between health literacy and depression among patients with hypertension. The results showed that health literacy was negatively related to depressive symptoms among study samples. Individuals with low health literacy often exhibit poor self-esteem, shame, and embarrassment (61), leading to social isolation and psychological barriers to asking for help, which may contribute to depressive symptoms among this group. Interestingly, our study evidenced the mediating effect of health literacy on the relationship between physical comorbidity and depression. Patients with hypertension and comorbidity may have a better understanding of different diseases, and thus comorbidity may be helpful in improving their health literacy and ultimately reducing their risk of depression. Health literacy is not only directly negatively related to depression but has also been shown to weaken the possible adverse effects of physical comorbidity on depression.

The mean social support score of our study sample was 37.17, and the proportion of individuals with a high level of social support was just 16.9%. Previous studies have reported that subjects with lower levels of social support are more likely to develop cardiovascular disease due to a history of hypertension and are at increased risk of experiencing higher blood pressure, less nocturnal blood pressure decrease, and a worse prognosis after a cardiovascular event (62–64). One study reported a threefold increase in the risk of all-cause mortality among patients with hypertension and poor social support (65). Therefore, the insufficient social support available to patients with hypertension deserves attention. Objective support and support utilization were relatively low in our sample, with scores of 7.91 ± 2.42 and 6.47 ± 2.08, respectively. Objective support refers to individual social networks and actual support received from a spouse, other family members, friends, relatives, workmates, work units, and party committees in the past, especially in times of distress and crisis. Rural hypertensive patients usually work in agriculture, and they lack financial support and support from workmates, work units, or party committees in solving practical problems, which may explain the low levels of objective support. In addition, physical activity and self-efficacy regarding physical exercise levels are generally low in patients with hypertension (66). This weakens their contact with social networks and thus reduces the social support potentially available to them. In this study, the utilization of social support by patients with hypertension was relatively low, consistent with other research (67). The degree of support utilization is associated with patients' compliance with anti-hypertensive therapy and blood pressure control (68). However, patients with hypertension are limited in their activities of daily living due to the illness, which leads to reduced social intercourse (69). This prevents them from making full use of support, even when sufficient resources are present, which is unfavorable in terms of addressing problems and causes negative emotions.

There is an extensive literature documenting that lack of social support is a strong predictor of depression among patients with hypertension (12–14). However, unlike other studies, our study showed that the direct relationship between social support and depression was not significant among study samples. Social support indirectly affected depression by the mediating effects of physical comorbidity and health literacy. A lack of social support was related to a greater likelihood of having multiple physical comorbidities, which may have been because social support can improve therapy adherence among patients with hypertension, thereby reducing the risk of developing other physical illnesses (39). In addition, that greater social support was associated with increased health literacy is consistent with a previous study (70). The mediating pathways suggested that lack of social support may have an impact on the increased risk of physical comorbidity and decreased health literacy, thus potentially leading to a higher prevalence of depression. Our research helps explain the mechanism by which social support influences depression and suggests interventions that may improve the mental health of patients with hypertension.

In addition, the linear regression revealed that gender and per capita annual family income were associated with depression among patients with hypertension. Compared with men, women had a higher degree of depression, consistent with previous studies of patients with hypertension (71, 72). Women have the dual responsibilities and pressures of family and work. Women with chronic disease experience difficulties fulfilling gender-specific social roles, thus increasing the risk of depression (73). In addition, depressive symptoms were fewer among high-income patients with hypertension than among those with low income. Lower income and poorer socioeconomic status have been confirmed as a risk factor for depression in patients with hypertension (74, 75). High-income individuals with hypertension can use more medical resources and health services and thereby obtain better disease control and psychological status (76).

Although our study adds important findings to the literature regarding the factors that influence depression and the mechanisms underlying these factors' relationships among patients with hypertension, we should acknowledge several limitations of this study. First, the cross-sectional design of this study enables the description of relationships between depression and social support, health literacy, and physical comorbidity; it does not enable one to infer causality of the three determinants on depression among individuals with hypertension. Second, this research was carried out in rural areas of Sichuan Province, limiting our ability to generalize the findings to other regions.

### Practice Implications

Overall, relieving depressive symptoms among individuals with hypertension requires the enhancement of social support and health literacy. In particular, more attention should be directed toward women, low-income individuals, and patients with physical comorbidities.

More community-based collective activities and social opportunities should be provided for individuals with hypertension, to address the lack of social support caused by disease-related barriers. Moreover, care providers are an important social support resource whose activities in this regard should be encouraged (77).

To enhance the health literacy of patients with hypertension, we suggest that health education programs should be developed to help this population improve their health knowledge and develop self-management behaviors. In addition, training in relaxation techniques or organizational skills for managing daily life activities should also help improve the mental health of patients with hypertension (14).

## Conclusions

Our findings indicated that social support, physical comorbidity, health literacy, gender, and per capita annual family income were significantly related to depression among patients with hypertension. Physical comorbidity had a direct positive relationship with depression, while health literacy was directly negatively associated with depression. Social support was indirectly negatively associated with depression in patients with hypertension, mediated by health literacy and physical comorbidity. Physical comorbidity had an indirect negative effect on depression via health literacy. In addition, female patients and patients with a per capita annual household income of <$750 had more depressive symptoms.

## Data Availability Statement

The raw data supporting the conclusions of this article will be made available by the authors, without undue reservation, to any qualified researcher.

## Ethics Statement

All of the participants signed an informed consent before investigation. The ethical approval of data collection was from the ethics committee of Sichuan University.

## Author Contributions

Conceptualization: DL and BZ. Methodology and funding acquisition: DL. Software, formal analysis, and writing—original draft preparation: BZ. Investigation: WZ, XS, and JG. Writing—review and editing: WZ, XS, JG, and DL. All authors contributed to the article and approved the submitted version.

## Conflict of Interest

The authors declare that the research was conducted in the absence of any commercial or financial relationships that could be construed as a potential conflict of interest.
